# Transition towards healthcare ‘net zero’: modelling condition-specific patient travel carbon emission estimations by transport mode in a retrospective population-based cohort study, Greater Glasgow, UK

**DOI:** 10.1136/bmjopen-2025-107016

**Published:** 2025-11-11

**Authors:** Jonathan R Olsen, Natalie Nicholls, Tran Quoc Bao Tran, Jill Pell, Jim Lewsey, Ruth Dundas, Jocelyn Friday, Clea Du Toit, Stefanie Lip, Daniel Mackay, Alan Stevenson, Richard Mitchell, Sandosh Padmanabhan

**Affiliations:** 1 Institute for Social Science Research, The University of Queensland, Brisbane, Queensland, Australia; 2 School of Health & Wellbeing, University of Glasgow, Glasgow, UK; 3 School of Cardiovascular & Metabolic Health, University of Glasgow, Glasgow, UK; 4 Digital Health Validation Lab, Living Lab, University of Glasgow, Glasgow, UK; 5 BHF Cardiovascular Research Centre, School of Cardiovascular and Metabolic Health, University of Glasgow, Glasgow, UK

**Keywords:** epidemiology, health services, health policy

## Abstract

**Abstract:**

**Objectives:**

To estimate condition-specific patient travel distances and associated carbon emissions across common chronic diseases in routine National Health Service (NHS) care, and to assess the potential carbon savings of modal shifts in transportation.

**Design:**

Retrospective population-based cohort study.

**Setting:**

NHS Greater Glasgow and Clyde, Scotland.

**Participants:**

6599 patients aged 50–55 years at diagnosis, including cardiovascular disease (n=1711), epilepsy (n=1044), cancer (n=716), rheumatoid arthritis (RA; n=172) and a matched control group based on age, sex and area-level deprivation (n=2956).

**Main outcome measures:**

Annual home-to-clinic distances and associated carbon emissions modelled under four transport modes (petrol car, electric car, bus, train) across five time points: 2-year prediagnosis, diagnosis year and 2-year postdiagnosis.

**Results:**

Mean annual travel distances to hospital varied by condition and peaked at diagnosis. Patients with cancer had the highest travel distances (161 km/patient/year for men; 139 km/patient/year for women), followed by RA (approximately 78 km/patient/year). The matched control group travelled <2 km/patient/year on average. Assuming 100% petrol car use, estimated condition-specific emissions ranged from 16.5 kg CO_2_/patient/year to 8.0 kg CO_2_/patient/year. Bus travel resulted in intermediate emissions, estimated between 10.5 and 8.0 kg CO_2_/patient. When travel was modelled using electric vehicles, emissions dropped between 3.5 and 2.7 kg for all conditions. Train travel produced similarly low emissions. Reducing petrol car travel from 100% to 60% lowered emissions up to 6.6 kg CO_2_/patient.

**Conclusions:**

Condition-specific estimates of healthcare-related travel emissions provide baseline understanding of the opportunities and challenges for decarbonising healthcare. Emission reduction is most achievable through modal shift, yet such shifts depend on factors beyond NHS control—such as transport infrastructure, digital access and social equity. Multisectoral strategies, including targeted telemedicine and integrated transport and urban planning, are critical to achieving net-zero healthcare while maintaining equitable access to care.

**STRENGTHS AND LIMITATIONS OF THIS STUDY**

- Large retrospective population-based cohort study including 6599 patients aged 50–55 years at diagnosis with comprehensive hospital visit data.
- Precise measurement of road network distances for all patient journeys across different clinical conditions and visit types.
- Robust carbon footprint estimates calculated using validated emission factors across multiple transport modes.
- Patient transport mode was estimated rather than directly observed, potentially affecting accuracy of carbon footprint calculations.
- Distance measurements may not reflect actual patient travel routes or transport choices.

## Introduction

 Climate change has been declared a public health emergency due to its significant risks to human health and well-being.^1^ At the 2015 United Nations Climate Change conference, 196 countries, including the UK, committed to the Paris Agreement, an international treaty aimed at reducing greenhouse emissions and limiting global heating to 1.5°C above pre-industrial levels.^2^ To achieve this target, the UK government has committed to reach ‘net zero’, meaning greenhouse gas (GHG) emissions will be balanced by those removed from the atmosphere.^3^

In Scotland, the National Health Service (NHS) has committed to being ‘net zero’ by 2045 at the latest.^4^ Patient travel contributes 5% of the NHS’s total carbon emissions, making it a significant focus for achieving net zero. During 2024, there were 1.5 million outpatient visits to secondary care medical specialties in Scotland,^5^ and it has been estimated that 90% of journeys to Scottish hospital sites are by car.^6^ In England, although the NHS’s overall carbon emissions decreased by 24% from 1990 to 2019, patient travel emissions rose by 95.2% during the same period, from 0.63 to 1.23 million metric tonnes of carbon dioxide equivalent,^7^ highlighting it as a critical target for intervention.

Achieving net-zero requires coordinated efforts across multiple levels, including patients, frontline services, hospital management and national and local governments.^8^ At the patient level, different transportation modes vary in carbon intensity, from active travel (walking, cycling) to public transport to fossil-fuel vehicles.^9^ At the clinical service level, the frequency of clinic visits directly influences travel-related emissions. National policies, such as transitioning to zero-emission vehicles, will also play a significant role.^10^

Carbon emissions vary by healthcare service type, with secondary care services contributing over half of total emissions compared with ambulance, community, primary care and non-clinical services.^7 11^ However, there is limited evidence on emissions related to patient travel by clinical specialty or condition. This information is vital for guiding targeted interventions across healthcare planning, delivery and management.

Cancer and cardiovascular disease (CVD) are leading causes of morbidity and mortality globally.^12^ Patients with these conditions often require frequent healthcare visits, diagnostic procedures and ongoing clinical management, making them significant contributors to healthcare-associated travel emissions.^13^ Cancer and CVD, among the most resource-intensive conditions, often require complex, multidisciplinary care. These diseases also disproportionately affect socioeconomically disadvantaged populations, raising important equity considerations when designing carbon-reduction strategies as the burden of interventions or service delivery changes would have a significant impact on this population group.^14 15^ Rheumatoid arthritis (RA) is a chronic inflammatory condition necessitating regular outpatient follow-up for disease monitoring and management,^16^ offering insights into the cumulative travel burden associated with sustained care. Epilepsy, a long-term neurological disorder, also involves ongoing specialist consultations,^17^ although typically with less intensive resource use than cancer or CVD, providing a valuable contrast in travel-related emissions across different disease profiles. Understanding how these conditions contribute to patient travel emissions will identify high-impact areas for targeted interventions aimed at achieving healthcare net-zero goals. Conditions like cancer and CVD are frequently diagnosed or become significantly more prevalent for the 50–55 years age group.^12^ This age group provides a clinically meaningful window, as it encompasses the onset of increased incidence for cancer and CVD, while avoiding age-related variability in healthcare needs and travel behaviours. Standardising a cohort in this way enables clearer comparisons of travel patterns and emissions across conditions and strengthens the relevance of findings for policy planning and equitable carbon reduction efforts in healthcare.

This study focuses on four conditions—RA, epilepsy, cancer and CVD—as well as a reference matched control group not undergoing clinical care or known multimorbidity to explore the differing patterns and impacts of patient travel. To enhance comparability and minimise confounding, the analysis is limited to patients aged 50–55 years.

The aims of this study are to:

1. calculate the home to clinic distances for patient travel to secondary care services in NHS Greater Glasgow and Clyde (NHS GGC);
2. explore variations in mean travel distance per patient by clinical conditions at five annual time points: 2 years prior to diagnosis, diagnosis and 2 years postdiagnosis;
3. estimate the mean carbon emissions per patient arising from travel to secondary care, by clinical condition and transportation mode.

## Methods

Record linkage of routine health data was used to conduct a population cohort study of people resident in NHS GGC. The NHS GGC region is located in the south-west of Scotland, an area covering 1150.7 km^2^ with a total population of 1.3 million people.^18 19^ The NHS GGC Health Board is responsible for providing and managing health services, including primary and secondary care, across this geographical area.

### Datasets and variables

The data extraction and linkage for this study were conducted by the NHS West of Scotland Safe Haven using a unique patient identifier—the Community Health Index (CHI). The data extract comprised de-identified patient records, including sociodemographic characteristics, hospital admissions with diagnostic and procedural codes and death records.

This study used linked data extracted from six datasets: the Prescribing Information System (PIS), the Scottish Morbidity Record 01 (SMR01, hospital admissions), the Scottish Morbidity Record 00 (SMR00, outpatient attendances), TrakCare Accident and Emergency (A&E) attendances, Scottish Care Information (SCI) Store (laboratory tests) and death certificates. The PIS collects data on all medications dispensed in the community, which are coded using the British National Formulary (BNF). The SMR01 collects data on hospital admissions, including the date of admission and diagnoses, which are recorded using International Classification of Diseases 10 (ICD-10) codes. The TrakCare A&E system collects arrival and discharge dates, and clinical diagnoses coded with ICD-10. SMR00 records date of attendance at NHS outpatient clinics and the clinical specialty. The Scottish SCI database collects laboratory test dates and results, as well as reference ranges. Death certificates record date and cause of death and are also coded using ICD-10. Demographic data included the patients’ age, sex (men/women) and Scottish Index of Multiple Deprivation (SIMD) quintile of the Scottish population. The SIMD is an area-based measure of deprivation, derived from aggregated Census data on employment, income, health, education, housing, crime and access to local services applied to 6976 distinct data zones of residence, with a mean average population size of 760 per data zone. A lower SIMD value corresponds to a higher level of socioeconomic deprivation.

### Data sources

**Figure 1:**
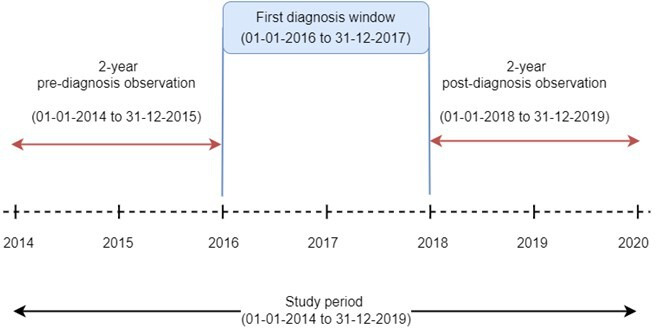
Study timeline. The diagram illustrates the overall study period (1 January 2014 to 31 December 2019). Patients were included if their first diagnosis occurred within the designated diagnosis window (1 January 2016 to 31 December 2017). This ensured each patient had a complete 2-year prediagnosis and 2-year postdiagnosis observation window within the study period for analysis.

The cohort comprised patients aged 50–55 years at diagnosis, residing within NHS GGC. The overall study period was 1 January 2014 to 31 December 2019. Longitudinal data were analysed across a structured observation window: a 2-year prediagnosis period (1 January 2014 to 31 December 2015), a diagnosis window (1 January 2016 to 31 December 2017) and a 2-year postdiagnosis period (1 January 2018 to 31 December 2019) (figure 1). Patients were included if they were diagnosed with one of four selected conditions: cancer, CVD, RA or epilepsy, during the diagnosis window and had no other comorbidity. This specific diagnosis window ensured each patient had the required 2-year prediagnosis and postdiagnosis data for analysis. The precise diagnostic criteria for RA and epilepsy, along with the specific diseases included under the umbrella terms of cancer and CVD, were ascertained through either an eligible ICD-10 diagnosis code recorded in SMR01 or eligible BNF-coded medication recorded in the PIS (online supplemental table 1). For each condition, the date of first diagnosis was defined as the earliest date on which an eligible diagnosis or medication code was recorded. All patients within the eligible study population who received an index diagnosis for the studied conditions during the defined study time frame were included in this study. A matched control group was also created: for each patient with an index condition, a corresponding control participant without the condition was selected from the same population. This control group was established using an exact matching methodology, pairing individuals based on age, sex and their home SIMD quintile. To ensure comparable observation periods, the diagnosis year of the case patient was assigned as the index year for their reference comparison patient.

### Total distance

All patient residential and clinic postcodes were geocoded. Individual-level home-to-clinic travel distances were calculated using NetworkX in Python (V.3.4.2) with the Ordnance Survey Open Roads dataset to capture realistic route-based (network) distances. The distances from patient home postcode to hospital postcode, as well as distances to various laboratories (relating to patient travel for diagnostic tests or treatment monitoring), were calculated for each patient’s visit during a single year and summed to get a total distance associated with that patient for each of the 5 years of follow-up. The included visits consisted of all scheduled clinic appointments and inpatient admissions after the initial diagnosis, covering periods of active treatment, such as radiotherapy and chemotherapy, as well as attendances for routine monitoring. For each patient’s visit, the distance was calculated using the specific address listed in the patient’s electronic health record at the time of that event. This ensures that any patient relocation during the study period is accurately reflected in our distance calculations. Where the distances between home and clinic were zero (eg, sharing of same postal code), the distances were set to 0.1 instead, as were patients registered at hospitals due to long stays. This total distance (in km) was used to derive the average annual patient carbon footprint by condition.

### Statistical analyses

Patient counts, total and average distances travelled were derived for each clinical condition; overall and stratified by sex. Because there were five annualised measurements of distance associated with clinical conditions for each patient (figure 1), generalised estimating equation (GEE) models were used, with yearly distances as the response variable, and clinical condition, time, SIMD quintile and distance between home and clinic set as independent variables. Time (in years) was treated as a continuous variable and included as a second-order polynomial to allow for a non-linear association with distance, and an interaction term between time and condition was fitted to determine whether distance patterns might vary between the conditions. The GEE models were run separately for men and women, calling the Poisson distribution due to the skew within the data. Other distributions considered were gamma (not valid due to presence of zeroes) and gaussian. For the latter, a suitable transformation was not found, and fitting the data as given produced a poorer model fit as judged using quintile-quintile plots.

### Carbon footprint estimation

Carbon emissions were estimated under four transport scenarios: petrol-engine medium sized car, electric medium sized car, local bus and train (light rail/tram). Emission rates per kilometre were sourced from the UK Government’s 2021 GHG Conversion Factors.^9^ Using predicted distances from the GEE models, emissions were modelled for each condition and time point, assuming different proportions (0%–100%) of journeys taken by each mode. Time point 0 (diagnosis year) was used as the primary comparator across scenarios.

All analyses were conducted in R V.4.3.0, using the gee, geepack, effects and ggplot2 packages. Spatial analyses were performed using Python V.3.10. Methods, code and data (subject to Safe Haven approval) are available on reasonable request.

### Patient and public involvement

Patients and the public were not involved in this study.

## Results

### Cohort characteristics

The final cohort included 6599 patients aged 50–55 years at diagnosis: 716 with cancer, 1711 with CVD, 172 with RA, 1044 with epilepsy and using exact matching, 2956 patients were identified as a comparison cohort control group. Sex was evenly distributed (3277 women and 3322 men) (table 1). The cohort was skewed towards socioeconomically deprived groups: 43% of patients (n=2813) resided in the most deprived SIMD quintile, compared with 16% (n=1031) in the least deprived.

**Table 1:** Patient counts by sex, area-level deprivation and condition

	Area-level deprivation (quintiles)					Total
	1—most deprived	2	3	4	5—least deprived	
Sex						
Women	1384	594	414	396	489	3277
Men	1429	577	407	367	542	3322
Clinical condition						
Control group	1257	532	373	342	452	2956
Cancer	265	123	93	112	123	716
CVD	698	310	207	198	298	1711
Epilepsy	527	176	131	88	122	1044
RA	66	30	17	23	36	172

CVD, cardiovascular disease; RA, rheumatoid arthritis.

### Travel distances by condition and time

Mean annual home-to-clinic travel distances varied substantially by clinical condition and peaked in the year of diagnosis (table 2). At diagnosis, patients with cancer had the lengthiest mean travel distances: 161.2 km per annum for men and 139.3 km per annum for women (time point 0). Patients with RA had the second-highest mean distances (78.2 km for women, 77.9 km for men). Patients with epilepsy and CVD showed more modest travel burdens (33.1–45.2 km), while referenced controls had minimal travel (1.1–1.5 km/year). Travel distances generally declined in the 2 years following diagnosis, particularly for cancer (from 161 km to 68.5 km in women, and to 70.5 km in men). For CVD, RA and epilepsy, postdiagnosis distances remained relatively stable.

**Table 2:** Summarised mean total annual distances travelled (km) per patient by sex and condition

Time point (years)	Mean total annual distances travelled (km): women	Mean total annual distances travelled (km): men
Cancer		
−2	18.3	16.7
−1	25.7	19.8
0	161.2	139.3
1	122.8	126.1
2	68.5	70.5
CVD		
−2	17.4	16.1
−1	19.9	16.7
0	45.2	44.7
1	36.1	35
2	31.1	27.6
Epilepsy		
−2	24.7	19.2
−1	30.4	23.4
0	33.1	34.3
1	34.5	35.7
2	33.2	29.4
RA		
−2	23.7	18.7
−1	41	25.6
0	78.2	77.9
1	78.5	72.5
2	70.8	57.1
Control group		
−2	1.1	0.9
−1	0.9	1
0	1.1	1.5
1	1.1	1.4
2	1	1.5

Time points: −2=2 years prediagnosis, –1=1 year prediagnosis, 0=diagnosis year, 1=1 year postdiagnosis, 2=2 years postdiagnosis. Full table with median, SEs and IQR for total annual distances travelled are shown in online supplemental table 2.

CVD, cardiovascular disease; RA, rheumatoid arthritis.

### Longitudinal analysis

**Figure 2:**
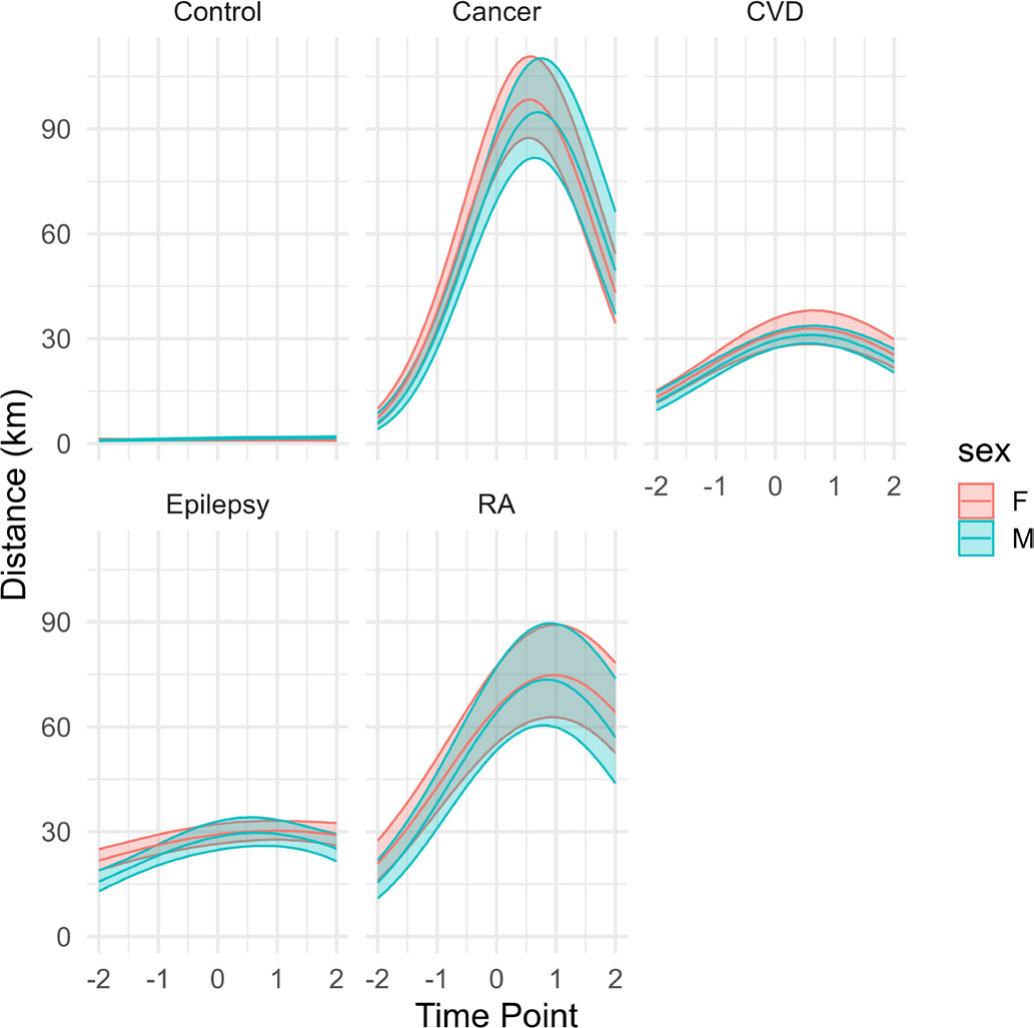
Associations between distance travelled and time for women and men by clinical group. Note: −2: 2 years prior to diagnosis; −1: 1 year prior to diagnosis; 0: year of diagnosis; 1: 1 year postdiagnosis; 2: 2 years postdiagnosis. CVD, cardiovascular disease; F, female; GEE, generalised estimating equation; M, male; RA, rheumatoid arthritis.

In GEE models (table 3), travel distance was significantly associated with clinical condition and time. Cancer diagnosis was associated with an 83-fold increase in annual travel distance for women (exp(β) 83.4; 95% CI 66.1 to 105.3) and 61-fold increase for men (exp(β) 61.4; 95% CI 45.6 to 82.6), compared with the matched control group. Significant non-linear trends over time in travel distances were observed for cancer, RA and CVD (p<0.001), which increased in the years up to diagnosis and then decreased in the years following, but not epilepsy (figure 2). Home-to-clinic distance was not associated with the frequency of visits, suggesting that proximity did not influence service use.

**Table 3:** Associations between annual distances travelled by time point (prediagnosis and postdiagnosis) and condition, controlling for distance from home and deprivation

		Exp (coef)	95% CI	P value	Exp (coef)	95% CI	P value
		Women			Men		
Condition	Control group	Ref			Ref		
	Cancer	83.4	66.1 to 105.3	<0.001	61.4	45.6 to 82.6	<0.001
	CVD	30.0	23.4 to 38.5	<0.001	23.0	17.2 to 30.7	<0.001
	Epilepsy	28.0	22.4 to 35.0	<0.001	22.2	16.4 to 30.2	<0.001
	RA	62.6	48.0 to 81.6	<0.001	49.9	35.7 to 69.6	<0.001
Time point	time	1.0	0.9 to 1.1	0.80	1.1	1.1 to 1.2	0.00
	time^2^	1.0	0.9 to 1.1	0.91	1.0	0.9 to 1.1	0.48
Area-level deprivation	Q1—most deprived	Ref			Ref		
	Q2	0.9	0.8 to 1.1	0.46	1.0	0.8 to 1.3	0.94
	Q3	0.8	0.7 to 1.0	0.06	1.0	0.9 to 1.3	0.73
	Q4	0.8	0.7 to 1.0	0.11	1.1	0.9 to 1.4	0.23
	Q5—least deprived	1.0	0.8 to 1.2	0.81	0.9	0.7 to 1.0	0.06
Interactions	Distance between home and clinic	1.1	1.1 to 1.1	<0.001	1.1	1.1 to 1.1	<0.001
	Cancer:time	1.6	1.4 to 1.8	<0.001	1.5	1.3 to 1.7	<0.001
	CVD:time	1.2	1.1 to 1.3	<0.001	1.0	1.0 to 1.1	0.37
	Epilepsy:time	1.1	1.0 to 1.2	0.06	1.0	0.9 to 1.1	0.82
	RA:time	1.3	1.2 to 1.5	<0.001	1.2	1.1 to 1.4	0.00
	Cancer:time^2^	0.7	0.6 to 0.7	<0.001	0.7	0.6 to 0.8	<0.001
	CVD:time^2^	0.9	0.8 to 0.9	<0.001	0.9	0.8 to 1.0	0.00
	Epilepsy: time^2^	1.0	0.9 to 1.0	0.31	0.9	0.9 to 1.0	0.13
	RA:time^2^	0.9	0.8 to 0.9	<0.001	0.9	0.8 to 0.9	<0.001

The table examines annual distances travelled by condition, time point and patients’ residential area-level deprivation, as well as interactions between these. Time point (in years) was treated as a continuous variable and included as a second-order polynomial to allow for a non-linear association with distance.

CVD, cardiovascular disease; RA, rheumatoid arthritis; Ref, reference.

### Estimated carbon emissions by clinical condition

**Figure 3:**
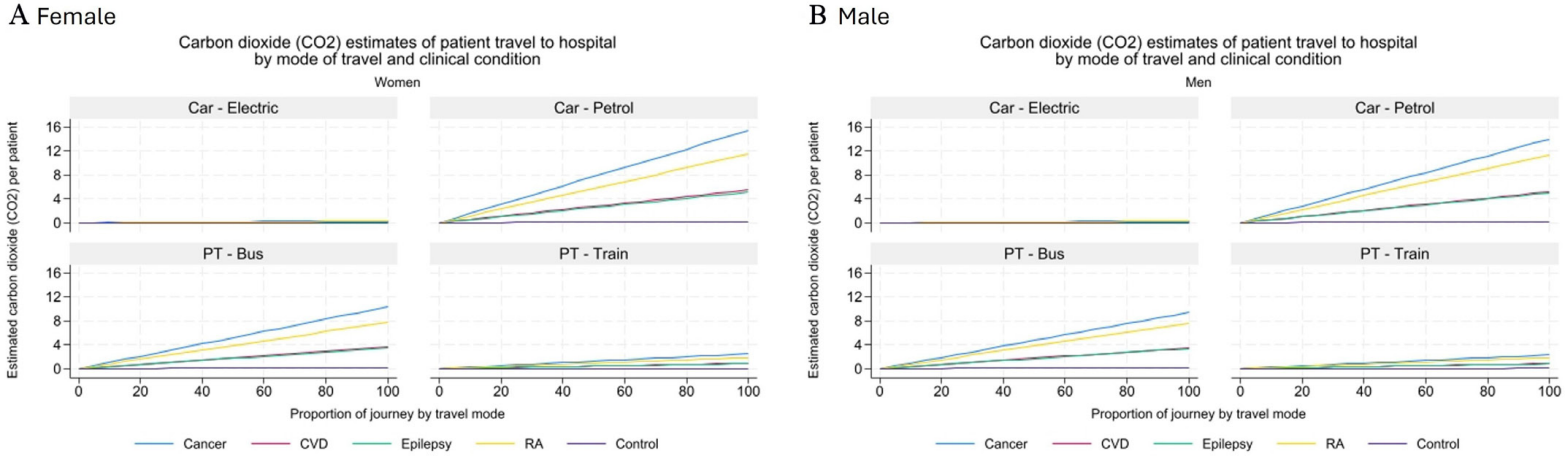
Carbon dioxide estimates of patient travel (PT) to hospital by mode of travel and clinical condition for (a) females & (b) males. Source: UK Goverment’s GHG Conversion, 2021. CVD, cardiovascular disease; RA, rheumatoid arthritis.

Figure 3A,B present the estimated carbon emissions per kilometre for various travel scenarios, assuming 0%–100% of the journey is completed using each of the four transport modes, stratified by condition and sex at the point of initial diagnosis (time point 0).

At 100% petrol car use, patients with cancer generated the highest emissions, estimated at 16.5 kg CO_2_/patient, followed by patients with RA (12.5 kg), CVD (10.5 kg) and epilepsy (8.0 kg). The lowest emissions were observed in the matched control group (0.5 kg). Assuming full travel by electric car, emissions were considerably lower across all groups—3.5 kg CO_2_/patient for cancer, 2.7 kg for RA and below 2 kg for epilepsy and CVD. Train travel produced similarly low emissions, ranging from 3.5 kg for cancer to 0.1 kg for the matched control group. Bus travel resulted in intermediate emissions, estimated at 10.5 kg CO_2_/patient for cancer and 8.0 kg for RA. Across all modes, emissions scaled linearly with the proportion of the journey completed. For example, reducing petrol car use from 100% to 60% for patients with cancer would lower emissions from 16.5 kg to 9.9 kg CO_2_/patient, a saving of 6.6 kg, while shifting to 60% electric car use would result in emissions of 2.1 kg CO_2_/patient. These findings highlight the significant mitigation potential associated with both transport electrification and modal shifts. Differences in emissions between conditions were most pronounced under petrol car scenarios and diminished substantially when sustainable modes were used. Notably, when using electric vehicles or trains, estimated emissions for all conditions fell within a relatively narrow range of 1.6–3.5 kg CO_2_/patient, indicating reduced condition-based variability.

## Discussion

### Key findings

This study highlights significant variation in patient travel distances and associated carbon emissions by clinical condition. Patients with cancer and RA had the highest travel burden, particularly around the time of diagnosis, with mean annual travel distances reaching 161 km for men with cancer and 139 km for women with cancer. These travel patterns translated into substantially higher carbon emissions when using petrol vehicles—up to 16.5 kg CO_2_ per patient for cancer and 12.5 kg for RA, compared with 10.5 kg for CVD and 8.0 kg for epilepsy. By contrast, emissions for individuals in the matched control group were negligible (0.5 kg).

While the number of patient visits is directly associated with the overall carbon footprint, the mode of transport has a substantial impact on emissions. Travel by petrol car has the largest carbon footprint per km and, therefore, contributed the largest absolute differences in carbon footprint between clinical conditions. Shifting to electric vehicles or public transport significantly reduced emissions across all groups, with electric vehicle use lowering cancer-related travel emissions to 3.5 kg CO_2_/patient and similar reductions observed for other conditions.

### Comparison with other literature

While prior literature has emphasised the growing impact of healthcare travel on NHS carbon emissions, few studies have explored condition-specific patterns. Our findings align with earlier reports highlighting the potential of telemedicine to reduce travel emissions in oncology and cardiovascular care. The differences in travel burden by condition are also consistent with known variations in care intensity and service pathways, particularly for cancer, where multiple treatment modalities and follow-up requirements contribute to frequent clinic attendance.

There are multiple opportunities to reduce patient travel to hospitals, including decreasing the frequency of clinic visits, improving proximity to healthcare services by moving services into communities and expanding the use of telemedicine.^15^ Additionally, modifications in clinical treatment pathways or surveillance protocols may reduce the need for secondary care visits.

The large majority of evidence from chronic disease management suggests significant environmental benefits from virtual consultations. For example, online consultations reduced patient travel by approximately 310 858 in-state miles and 188 346 out-of-state miles at an academic medical centre, leading to substantial reductions in fuel consumption and emissions.^20^ Teleconsultations offer patients a flexible and safe alternative for outpatient reviews while substantially reducing healthcare-related carbon emissions.^21^ Evidence from gastroenterology suggests that shifting some consultations to telehealth does not negatively impact clinical outcomes, although it may lead to an increase in clinician-ordered blood tests.^22^

Telemedicine has demonstrated substantial potential in managing CVD, such as heart failure and coronary artery disease, by significantly reducing the requirement for in-person clinic visits. For instance, telemedicine reduced carbon emissions from patient travel from 9.77 kg CO_2_e to 0.41 kg CO_2_e per consultation, as observed in a study conducted in Italy.^23^ For CVD, there was a reduction of 41.2 kg CO_2_e per patient if follow-up was remote.^24^ A systematic review further highlighted telemedicine’s effectiveness in decreasing hospitalisations and emergency department visits, demonstrating virtual consultations’ equivalence or superiority compared with traditional care.^25 26^ The use of remote patient monitoring (RPM) and telenursing programmes further supports the management of CVD. A Virginia-based programme reported a 65% decrease in cardiovascular hospital admissions through RPM, significantly reducing healthcare costs and travel-associated carbon footprints.^27^ Furthermore, telemedicine optimises care pathways, shortening wait times for in-person visits for conditions like chronic coronary syndrome by nearly 3 months.^28^ Integration of wearable technologies for telemonitoring has also improved early detection of acute heart failure decompensation, reducing unnecessary travel.^29^

We found that patients with cancer had the highest mean annual distance travelled. This is likely due to clinical oncology including diagnosing and treating cancer, as well as chemotherapy, radiotherapy and other systemic therapies, which require frequent hospital visits. In Scotland during 2024, 87% of the 139 448 outpatient clinical oncology clinic visits were follow-up.^5^ While some consultations inevitably require physical contact, such as the administration of radiotherapy or chemotherapy, the adoption of telemedicine within oncology has notably reduced environmental impacts associated with cancer care. In Scotland, cancer follow-up appointments and postoperative care consultations are offered in person or by phone.^30^ In addition, Dana-Farber Cancer Institute reported an 81.3% reduction in GHG per telemedicine visit compared with face-to-face consultations, resulting in an estimated annual reduction of approximately 75.3 million kg CO_2_e.^31^ Similarly, a National Cancer Institute-designated centre estimated savings of 424–471 kg CO_2_ emissions per telemedicine visit for patients within a 60 min drive, equivalent to removing 91.5 passenger vehicles from the road annually.^32^ In Manitoba, Canada, virtual cancer care saved approximately 87 000 to 155 000 kg CO_2_ emissions monthly, reinforcing telemedicine’s broader environmental benefits.^33^

Although specific research on telemedicine for RA remains limited, data from chronic disease management suggest environmental benefits from virtual consultations are likely.^20^ Telemedicine has proven effective in epilepsy care, significantly decreasing carbon emissions by reducing patient travel. A UK-based study noted substantial carbon emission savings over 6 months, primarily due to decreased travel distances. Emissions from telemedicine infrastructure were negligible relative to the savings achieved.^34^ These results underscore telemedicine’s viability as an environmentally sustainable solution in epilepsy management.

Achieving zero-emission healthcare requires sustainable, adaptable and efficient strategies that maintain the delivery of safe, high-quality care.^22^ The NHS Scotland Climate Emergency and Sustainability Strategy outlines plans to minimise patient and visitor travel through adoption of 20 min neighbourhood principles in health facility planning and promotion of integrated care models to reduce separate appointments and associated journeys.^35^ However, this approach may conflict with evidence supporting high-quality specialist centres, which have demonstrated improved patient outcomes.^36^ Furthermore, only 51% of Scottish households are located within a 10 min walking distance of a community healthcare centre, highlighting potential accessibility challenges.^37^

Additional barriers include technological infrastructure requirements, particularly reliable internet connectivity and accessible devices in rural or underserved regions.^26 38^ Digital literacy barriers, reimbursement concerns and user acceptance must also be addressed to facilitate telemedicine adoption.^39 40^ Long-term research is needed to confirm telemedicine’s efficacy across diverse clinical scenarios and identify potential limitations.^25 41 42^

We recommend future studies examine a range of clinical service delivery options that could reduce patient carbon emissions, including relocating specialist clinics to community settings, expanding primary care-delivered services, implementing remote consultations and improving patient education to reduce unnecessary visits. Such investigations should consider the impact on clinical outcomes, patient safety, technological accessibility and patient acceptability. Additionally, we suggest modelling the carbon emission implications of these strategies for the NHS as a whole.

In addition to reducing travel frequency, changes in transport mode can significantly impact carbon emissions. Our findings indicate that increased use of public transport substantially reduces emissions compared with petrol engine private vehicle use. However, access to hospitals via public transport is not equitable and varies significantly across urban and rural settings.^43^ While patient travel using electric vehicles presents the greatest potential for reducing carbon emissions without decreasing clinic visits, access to electric vehicles remains inequitable. Electric vehicle ownership is predominantly concentrated among higher-income, highly educated individuals, typically homeowners with access to multiple vehicles and private charging infrastructure.^44^ This highlights that effective strategies for reducing carbon emissions from patient travel require multisectoral approaches—including the NHS and government-led initiatives.

### Strengths and limitations

This study draws on a comprehensive, population-level cohort from NHS GGC, representing a population of 1.3 million people, meaning the results are likely generalisable to other major urban areas in the UK. A major strength lies in the precise geospatial modelling of patient travel using real-world road networks, providing more accurate distance estimates than traditional straight-line (Euclidean) distances, which do not reflect actual travel routes. Our analysis also does not account for the journey to or from a public transit stop that may be on foot and assumes the whole journey was by a transport single mode. By modelling emissions across multiple transport modes and time points, the study provides a flexible and policy-relevant framework that can inform decarbonisation strategies tailored to clinical context. Limitations include the absence of individual-level data on actual transport modes, fuel type or specific patient behaviours (eg, trip chaining or caregiver travel), which limits precision in estimating emissions. Although we modelled a range of modal scenarios, these remain assumptions in the absence of travel survey validation. We used the UK Government’s 2021 GHG Conversion Factors to calculate emissions associated with patient journeys from home to clinic. However, these factors only capture use-phase emissions and do not include vehicle production emissions or other lifecycle factors associated with the transition to net zero. Additionally, the standard conversion factors may not reflect local variations in fleet composition, such as the increasing deployment of electric buses in urban areas. This may underestimate the true carbon impact of proposed interventions, particularly those involving electric vehicle adoption, where manufacturing emissions represent a substantial component of the total carbon footprint. Additionally, the analysis does not include primary care or community-based consultations, potentially underestimating the full carbon burden for some conditions. The four clinical conditions included in the study were chosen based on consultation with clinicians to examine our outcomes across a range of contrasting care patterns. Future studies should explore these outcomes for a broader range of conditions, including those with higher population-level morbidity and mortality. Finally, patients and the public were not involved in the design or interpretation of this study, although future work will aim to co-produce low-carbon care strategies with diverse stakeholder input.

## Conclusion

Condition-specific travel patterns generate substantial variation in healthcare-related carbon emissions. While emission reductions are achievable through modal shifts to electric vehicles, low-carbon or electric public transport, these solutions lie largely outside the direct remit of the NHS. Our findings highlight the need for a coordinated, cross-sectoral response that integrates sustainable transport infrastructure, digital-first care models and targeted investment in telemedicine—particularly around diagnosis periods when travel peaks. To ensure that decarbonisation efforts do not exacerbate existing health inequalities, carbon reduction strategies must consider socioeconomic barriers to electric vehicle access, public transport availability and digital connectivity. Equitable implementation will require tailored approaches sensitive to local context, particularly for socioeconomically deprived populations who may face greater challenges in adopting low-carbon options. Achieving a net-zero healthcare system demands more than technical solutions—it calls for system-wide innovation, political will and the embedding of environmental sustainability into clinical care pathways. By quantifying condition-specific travel emissions, this study offers a foundation for targeted action, supporting a greener NHS that maintains high-quality, accessible care for all.

## Supplementary material

10.1136/bmjopen-2025-107016 online supplemental file 1

## Data Availability

Data may be obtained from a third party and are not publicly available.
