# Supplementary material for: Transition towards healthcare ‘net zero’: modelling condition-specific patient travel carbon emission estimations by transport mode in a retrospective population-based cohort study, Greater Glasgow, UK
Source: BMJ Open. 2025 Nov 11;15(11):e107016. doi: 10.1136/bmjopen-2025-107016 (PMC12606482; doi:10.1136/bmjopen-2025-107016)
Supplement: online supplemental file 1 [file bmjopen-15-11-s001.docx]

**Transition towards healthcare ‘net zero’: Modelling condition-specific patient travel carbon emission estimations by transport mode: a retrospective population-based cohort study, Greater Glasgow, UK.**

Supplementary Material

***Supplementary Table 1: Multiple Long-Term Conditions Classification***

| **Group** | **Condition** | ***Inclusion criteria*** | **ICD codes** | **Medications** |
| --- | --- | --- | --- | --- |
| Cancer | Upper airway | 1st Hospitalisation in SMR01  AND  Attending oncology clinic >=1 time/year | C00-C14 |  |
| Cancer | GIT | 1st Hospitalisation in SMR01  AND  Attending oncology clinic >=1 time/year | C15-C26 |  |
| Cancer | Respiratory | 1st Hospitalisation in SMR01  AND  Attending oncology clinic >=1 time/year | C30-C39 |  |
| Cancer | Bone | 1st Hospitalisation in SMR01  AND  Attending oncology clinic >=1 time/year | C40-C41 |  |
| Cancer | Skin | 1st Hospitalisation in SMR01  AND  Attending oncology clinic >=1 time/year | C43-44 |  |
| Cancer | Mesothelial | 1st Hospitalisation in SMR01  AND  Attending oncology clinic >=1 time/year | C45-C49 |  |
| Cancer | Breast | 1st Hospitalisation in SMR01  AND  Attending oncology clinic >=1 time/year | C50 |  |
| Cancer | Female genital | 1st Hospitalisation in SMR01  AND  Attending oncology clinic >=1 time/year | C51-C58 |  |
| Cancer | Male genital | 1st Hospitalisation in SMR01  AND  Attending oncology clinic >=1 time/year | C60-C63 |  |
| Cancer | Urinary tract | 1st Hospitalisation in SMR01  AND  Attending oncology clinic >=1 time/year | C64-C68 |  |
| Cancer | CNS | 1st Hospitalisation in SMR01  AND  Attending oncology clinic >=1 time/year | C69-72 |  |
| Cancer | Thyroid/Endocrine | 1st Hospitalisation in SMR01  AND  Attending oncology clinic >=1 time/year | C73-C75 |  |
| Cancer | Neuroendocrine | 1st Hospitalisation in SMR01  AND  Attending oncology clinic >=1 time/year | C7A, C7B |  |
| Cancer | Haemological | 1st Hospitalisation in SMR01  AND  Attending oncology clinic >=1 time/year | C81-C96 |  |
| Addiction | Alcohol misuse | 1st Hospitalisation in SMR01  or  Prescription of an alcohol dependence treatment | E52, F10, G62.1, I42.6,K29.2, K70.0, K70.3, K70.9, T51, Z50.2, Z71.4, Z72.1 | **Alcohol dependence medications**: Disulfiram Acamprosate Nalmefene |
| Blood | Anaemia | 1st Hospitalisation in SMR01 | D55-D58, D60, D61, |  |
| Blood | Coagulation disorders | 2nd Hospitalisation in SMR01 | D65-D69 |  |
| CV | Arrhythmia | 1st Hospitalisation in SMR01  OR  Commencement of antiarrhythmic medications | I44-I49, except I48 | **Antiarrhythmic medications:** Amiodarone hydrochloride Disopyramide Disopyramide phosphate Flecainide acetate Mexiletine hydrochloride Procainamide hydrochloride Propafenone hydrochloride Quinidine sulfate Digoxin |
| CV | Atrial fibrillation | 1st Hospitalisation in SMR01  OR  >= 3 concomitant dispenses of an AF medication and an oral anticoagulant | I48.0 | **AF medication:** Bisoprolol fumarate Flecainide acetate Amiodarone hydrochloride Digoxin Dronedarone hydrochloride  **Oral anticoagulants:** Acenocoumarol Apixaban Dabigatran etexilate Edoxaban Phenindione  Rivaroxaban Warfarin sodium |
| CV | Cardiomyopathy | 1st Hospitalisation in SMR01 | I42, I43 |  |
| CV | Chronic heart failure | 1st Hospitalisation in SMR01 | I50 |  |
| CV | Hypertension | Initiation of antihypertensive therapy preceding CHF/CVD | I10-I13, I15 | **Antihypertensives:** Alpha blockers ACE inhibitors ARBs CCBs Centrally acting antihypertensives Loop diuretics K-sparing and aldosterones K-sparing diuretics Thiazides and related diuretics Vasodilator antihypertensives |
| CV | Myocardial infarction | 1st Hospitalisation in SMR01 | I21-I22 |  |
| CV | Peripheral vascular disease | 1st Hospitalisation in SMR01 | I70.2, I73 |  |
| CV | Stroke or TIA | 1st Hospitalisation in SMR01 | G45.0-G45.3, G45.8-G45.9, I60, I61, I63, I64 |  |
| CV | Congenital heart dieases | 1st Hospitalisation in SMR01 | Q20-Q28 |  |
| CV | Valvular heart disease | 1st Hospitalisation in SMR01 | I34-I37 |  |
| CV | Thromboembolism | 1st Hospitalisation in SMR01  AND  Oral anticoagulant for the next 3 months | I81-I82 | **Oral anticoagulants:** Acenocoumarol Apixaban Dabigatran etexilate Edoxaban Phenindione Rivaroxaban Warfarin sodium |
| Endocrine | Diabetes | 1st Hospitalisation in SMR01 | E10-E14 |  |
| Endocrine | Hypothyroidism | 1st Hospitalisation in SMR01  OR  1st prescription of a hypothyroidism medication | E00–E03, E89.0 | **Hypothyroidism medications:** Levothyroxine sodium Levothyroxine sodium and liothyronine Liothyronine sodium |
| Endocrine | Obesity | 1st Hospitalisation in SMR01 | E65-E68 |  |
| GI | Inflammatory bowel disease | (1st Hospitalisation in SMR01  AND   1st dispenses of sulfasalazine/mesalazine)  OR  prescription of sulfasalazine/mesalazine for > 3 months | K50, K51 | Balsalazide sodium Mesalazine Olsalazine sodium Sulfasalazine |
| GI | Irritable bowel syndrome | 1st Hospitalisation in SMR01 | K58  Exclusion: C18-C21, C25, C56, C78.5, C79.6, D01.7, D01.9, D37.1-D37.5, K50-K51, K70.2-K70.3, K74.0, K74.2, K74.6, K86.0-K86.1, K90, K91.2 |  |
| GI | Peptic ulcer disease | >=1 hospitalisation in 2 consecutive years in SMR01  OR  1st prescription of of PPI or H2 antagonist for >= 3 months | K25.7, K25.9, K26.7, K26.9, K27.7, K27.9, K28.7, K28.9 | Proton pump inhibitors  H2 antagonists |
| GI | Severe constipation | >=1 hospitalisation in 2 consecutive years in SMR01 | K55.8, K56.0, K56.4, K56.7, K59.0, K63.1, K63.4, K63.81, K63.88, K92.80, K92.88   Exclusion: C17-C21, C45.1, C48, C51-C58, C60-C68, C78.5-C78.6, D01.7, D01.9, D37.1-D37.5, K50-K51, K66.0, N73.6, N99.4 (K56.6 if R10.1) |  |
| Infection | Chronic viral hepatitis B | 1st Hospitalisation in SMR01  OR  Hep B surface antigen positive in SCI Store | B16, B18.0-B18.1 |  |
| Infection | Chronic viral hepatitis C | 1st Hospitalisation in SMR01  OR  Hep C RNA positive in SCI Store | B18.2 |  |
| Infection | Human immunodeficiency virus (HIV) disease | 1st Hospitalisation in SMR01  OR  HIV antibody/antigen positive   OR   Presence of lab tests for HIV antibody/antigen and CD4/CD8 multiple times in >=2 consecutive years | B20 |  |
| KUB | AKI/CKD | 1st Hospitalisation in SMR01 | N17-N19 |  |
| KUB | Glomerular/Tubular | 1st Hospitalisation in SMR01 | N00-N16 |  |
| KUB | Prostate | 1st Hospitalisation in SMR01 | N40-N42 |  |
| Liver.GB.Panc | Chronic Liver | 1st Hospitalisation in SMR01 | K70-K77 |  |
| Liver.GB.Panc | GB diseases | 1st Hospitalisation in SMR01 | K80-K83 |  |
| Liver.GB.Panc | Pancreas | 1st Hospitalisation in SMR01 | K85, K86 |  |
| MentalHealth | Intellectual disabilities | 1st Hospitalisation in SMR01 | F70-F79 |  |
| MentalHealth | Developmental | 1st Hospitalisation in SMR01 | F80-F89 |  |
| MentalHealth | Schizophrenia/Psychosis | 1st Hospitalisation in SMR01   OR  Dispenses of antipsychotics at least once per year for >=2 years | F20-F29 | Antipsychotics |
| MentalHealth | Anxiety | 1st Hospitalisation in SMR01   OR  Dispenses of anxiolytics at least once per year for >=2 years | F40-F48 | Anxiolytics |
| MentalHealth | personality | 1st Hospitalisation in SMR01 | F60-F69 |  |
| MentalHealth | Dementia | 1st Hospitalisation in SMR01 | F00–F03, F05.1, G30, G31.1 |  |
| MentalHealth | Depression | 1st Hospitalisation in SMR01   OR  Dispenses of antidepressants at least once per year for >=2 years | F20.4, F31.3–F31.5, F32, F33, F34.1, F41.2, F43.2 |  |
| MSK | Rheumatoid arthritis | 1st Hospitalisation in SMR01   OR  concomitant dispenses of 2 or more of hydroxychloroquine/methotrexate/NSAID/ leflunomide/sulfasalazine | M05, M06, M31.5, M32–M34, M35.1, M35.3, M36.0 | Hydroxychloroquine sulfate Methotrexate NSAIDs Leflunomide Sulfasalazine |
| MSK | Arthropathy | 1st Hospitalisation in SMR01   OR  >=1 dispense of rheumatic disease medication for > 1 year | M00-M25 | Drugs used in rheumatic diseases and gout |
| MSK | Spondylopathies and dorsopathies | 1st Hospitalisation in SMR01 | M45-M49 |  |
| MSK | Osteoporosis | 1st Hospitalisation in SMR01   OR  Commencement of bisphosphonates | M80-M85 | **Bisphosphonates:**  Alendronic acid Etidronate disodium Ibandronic acid Pamidronate disodium Risedronate sodium Sodium clodronate Strontium ranelate Tiludronic acid Zoledronic acid |
| Neurology | Chronic pain | 1st Hospitalisation in SMR01   OR  Opioid analgesics, or non-opioid analgesics, or treatment for neuropathic pain for at least 3 months/year for >= 3 years | F45.4, M08.1, M25.50, M25.51, M25.55 - M25.57, M43.2 - M43.6, M45, M46.1, M46.3, M46.4, M46.9, M47, M48.0, M48.1, M48.8, M48.9, M50.8, M50.9, M51, M53.1 - M53.3, M53.8, M53.9, M54, M60.8, M60.9, M63.3, M79.0 - M79.2, M79.6, M79.7, M96.1 | Opioid analgesics Non-opioid analgesics Neuropathic pain medication |
| Neurology | Epilepsy | 1st Hospitalisation in SMR01   OR  >=1 dispense of antiepileptic for > 1 year | G40-G41 | Antiepileptics |
| Neurology | Multiple sclerosis | 2 Hospitalisations in SMR01 | G35, G36, G37, H46 |  |
| Neurology | Parkinson’s disease | 1st Hospitalisation in SMR01   OR  >=1 dispense of Parkinson treatment for > 1 year | G20, G21, G22 | Parkinson medications |
| Neurology | Inflammatory | 1st Hospitalisation in SMR01 | G00-G09 |  |
| Neurology | Other degenerative diseases | 1st Hospitalisation in SMR01 | G10-G32, except G20-G22 |  |
| Neurology | Neuropathy | 1st Hospitalisation in SMR01 | G50-G65 |  |
| Neurology | Muscle | 1st Hospitalisation in SMR01 | G70-G73 |  |
| Neurology | Paralytic/palsy | 1st Hospitalisation in SMR01 | G80-G83 |  |
| Pelvic | Female pelvic diseases | 1st Hospitalisation in SMR01 | N70-N98 |  |
| Pelvic | Male genital | 1st Hospitalisation in SMR01 | N43-N53 |  |
| Respiratory | COPD/asthma | 1st Hospitalisation in SMR01 | J40-J47 |  |
| Respiratory | External agents | 1st Hospitalisation in SMR01 | J60-J70 |  |
| Respiratory | Pleural | 1st Hospitalisation in SMR01 | J90-J94 |  |
| Respiratory | Infiltrative diseases | 1st Hospitalisation in SMR01 | J82, J84 |  |
| Skin | Psoriasis | 1st Hospitalisation in SMR01   OR  >=1 dispense of psoriasis preparations | L40.0 - L40.4, L40.8, L40.9 | Psoriasis preparations |
| Skin | Bullous | 1st Hospitalisation in SMR01 | L10-L14 |  |
| Skin | Dermatitis | 1st Hospitalisation in SMR01 | L20-L30 |  |
| Skin | Papulosquamous | 1st Hospitalisation in SMR01 | L41-L45 |  |

***Supplementary Table 2: Summarised total annual distances travelled (km) per patient by sex and condition***

| **Time point** | **mean** | **se** | **median** | **25th** | **75th** | **mean** | **se** | **median** | **25th** | **75th** |
| --- | --- | --- | --- | --- | --- | --- | --- | --- | --- | --- |
|  | **Women - annual distances travelled (km)** | | | | | **Men - annual distances travelled (km)** | | | | |
| **Cancer** | | | | | | | | | | |
| -2 | 18.3 | 1.9 | 6.8 | 0.0 | 21.8 | 16.7 | 2.0 | 4.5 | 0.0 | 17.3 |
| -1 | 25.7 | 4.0 | 7.5 | 0.0 | 25.8 | 19.8 | 2.2 | 8.0 | 0.0 | 25.5 |
| 0 | 161.2 | 9.9 | 95.1 | 42.8 | 195.5 | 139.3 | 9.4 | 78.5 | 36.7 | 176.3 |
| 1 | 122.8 | 9.3 | 52.2 | 12.0 | 148.4 | 126.1 | 13.1 | 40.6 | 8.3 | 106.9 |
| 2 | 68.5 | 7.1 | 21.9 | 0.0 | 64.5 | 70.5 | 10.1 | 15.0 | 0.0 | 53.7 |
| **CVD** | | | | | | | | | | |
| -2 | 17.4 | 1.2 | 8.1 | 0.0 | 20.9 | 16.1 | 1.8 | 4.1 | 0.0 | 15.6 |
| -1 | 19.9 | 1.5 | 9.3 | 0.0 | 23.0 | 16.7 | 1.3 | 5.3 | 0.0 | 17.2 |
| 0 | 45.2 | 3.3 | 27.1 | 12.2 | 56.9 | 44.7 | 1.8 | 26.8 | 11.3 | 54.7 |
| 1 | 36.1 | 4.1 | 16.7 | 6.0 | 42.1 | 35.0 | 1.9 | 16.9 | 5.1 | 39.4 |
| 2 | 31.1 | 2.8 | 14.8 | 3.9 | 35.6 | 27.6 | 2.0 | 11.3 | 3.2 | 32.5 |
| **Epilepsy** | | | | | | | | | | |
| -2 | 24.7 | 1.9 | 11.6 | 3.0 | 27.2 | 19.2 | 1.6 | 8.1 | 0.0 | 21.6 |
| -1 | 30.4 | 2.0 | 15.6 | 3.9 | 37.9 | 23.4 | 1.9 | 9.9 | 0.6 | 27.7 |
| 0 | 33.1 | 1.8 | 19.5 | 7.6 | 41.2 | 34.3 | 3.7 | 18.0 | 5.9 | 40.6 |
| 1 | 34.5 | 2.0 | 17.7 | 7.3 | 39.2 | 35.7 | 4.1 | 15.9 | 4.7 | 38.8 |
| 2 | 33.2 | 1.8 | 18.3 | 6.3 | 42.9 | 29.4 | 2.1 | 14.7 | 4.2 | 38.9 |
| **RA** | | | | | | | | | | |
| -2 | 23.7 | 3.5 | 11.4 | 1.4 | 28.6 | 18.7 | 3.0 | 10.0 | 0.0 | 26.8 |
| -1 | 41.0 | 7.5 | 17.8 | 5.9 | 35.6 | 25.6 | 4.8 | 13.3 | 4.2 | 31.3 |
| 0 | 78.2 | 7.9 | 51.7 | 27.3 | 94.5 | 77.9 | 11.2 | 52.4 | 25.2 | 90.2 |
| 1 | 78.5 | 8.6 | 49.1 | 26.3 | 94.5 | 72.5 | 8.1 | 70.8 | 21.0 | 105.8 |
| 2 | 70.8 | 8.0 | 45.2 | 22.7 | 95.4 | 57.1 | 8.7 | 34.0 | 13.8 | 75.4 |
| **Control Group** | | | | | | | | | | |
| -2 | 1.1 | 0.1 |  |  |  | 0.9 | 0.1 |  |  |  |
| -1 | 0.9 | 0.1 |  |  |  | 1.0 | 0.1 |  |  |  |
| 0 | 1.1 | 0.1 |  |  |  | 1.5 | 0.3 |  |  |  |
| 1 | 1.1 | 0.1 |  |  |  | 1.4 | 0.2 |  |  |  |
| 2 | 1.0 | 0.1 |  |  |  | 1.5 | 0.2 |  |  |  |

*Note: CVD = cardiovascular disease, RA = rheumatoid arthritis. The median and IQR for the control group were zero for all time points. SE = standard error. Time points: -2 = 2-years pre-diagnosis, –1 = 1-year pre-diagnosis, 0 = diagnosis year, 1 = 1-year post-diagnosis, 2 = 2-years post-diagnosis.*
