# Supplementary material for: Transition towards healthcare ‘net zero’: modelling condition-specific patient travel carbon emission estimations by transport mode in a retrospective population-based cohort study, Greater Glasgow, UK
Source: BMJ Open. 2025 Nov 11;15(11):e107016. doi: 10.1136/bmjopen-2025-107016 (PMC12606482; doi:10.1136/bmjopen-2025-107016)
Supplement: Supplementary file 2 [file bmjopen-2025-107016.reviewer_comments.pdf]

PEER REVIEW HISTORY

BMJ Open publishes all reviews undertaken for accepted manuscripts. Reviewers are asked to complete a checklist review form and are provided with free text boxes to elaborate on their assessment. These free text comments are reproduced below.

ARTICLE DETAILS

Title (Provisional)

Transition towards healthcare ‘net zero’: Modelling condition-specific patient travel carbon emission estimations by transport mode: a retrospective population-based cohort study, Greater Glasgow, UK

Authors

Olsen, Jonathan R; Nicholls, Natalie; Tran, Tran Quoc Bao; Pell, Jill; Lewsey, Jim; Dundas, Ruth; Friday, Jocelyn; Du Toit, Clea; Lip, Stefanie; Mackay, Daniel; Stevenson, Alan; Mitchell, Richard; Padmanabhan, Sandosh

---

VERSION 1 - REVIEW

**Reviewer**	**1**
**Name**	**Kühl, Susanne**
**Affiliation**	**Ulm University, Biochemistry and Molecular Biology**
**Date**	**01-Aug-2025**
**COI**	**None**

The study of Olsen et al. extimates the patient travel carbon emission across common chronic diseases and suggests carbon saving by alternative transport possibilities. To do end, data from 6,599 patients aged between 50 and 55 were ananlyzed retrospectively for three different periods. Cancer patients traveled the longest followed by RA patients. Travelling with electric vehicles and train have the lowest emission. To achieve a net-zero healthcare, the authors propose mulitsectoral strategies such as integrated transport planning and targeted telemedicine.

Even if the results are not suprising, they are stoll are important and should be of interest for a broad readership of BMJ Open. Some revisions should be made before publication.

General comment

Please explain all abbreviations when they are first used, including NHS.

The abstract provides a good overview about the study and the methods chapter is understandable.

Results:

Table 2 contains a relatively large amount of data and is therefore confusing. Is all the data relevant for the readers in the main manuscript? Would it be possible to reduce the data in the main tables and include the detailed data in the Supplement?

Starting on page 11 line 58 / 59: „Significant non-linear trends over time were observed for cancer, RA, and CVD (p<0.001), but not epilepsy (Figure 2).“ => What is the trend? It is rising or falling? The most important findings should be unterstable without looking at the figures and tables.

Figure 2: Consider a different structure for the figure, in which all graphs are displayed in a single line for each gender and the x-axes are not divided. One option is to arrange the data by high (women at the top, men at the bottom). All abbreviations should be explained in the figure legend (e.g. GEE...).

The dicussion mainly advocates a greater focus on telemedicine. To support this hypothesis, you should calculate the carbon emission of one telemedine consultation compared to the various transport options.

**Reviewer**	**2**
**Name**	**Carradice, Daniel**
**Affiliation**	**Hull York Medical School**
**Date**	**11-Aug-2025**
**COI**	**None**

Overall

I wish to congratulate the authors. This is an important topic and I find their work and the paper very interesting – I enjoyed reading it.

I think this does add valuable insights and data into this debate. I think it would benefit from some revision. It reads a bit like an exercise to qualify travel and see how much CO2 we could save by making everyone have an electric car. The situation is more complex than this and the solutions much more wide ranging. The authors do expand to accept this, but quite late in the paper and this consideration is not apparent in the design or main substance. I really look forward to seeing where they could take this next and there is clear potential impact here.

Title

These data are very specific to the geography, health service design and healthcare use in a specific population: Consider reflecting this in the title and manuscript.

I think the title does underplay what you are trying to achieve here. You are looking at specific actionable opportunities to reduce emissions.

Abstract

Participants: suggest listing cardiovascular first as largest group then in order of descending size followed by controls.

Introduction

Page 6

You state "Cancer and cardiovascular disease (CVD) are leading causes of morbidity and mortality globally". Cardiovascular disease is both more common and a greater cause of morbidity and mortality than cancer – consider reversing the order here and throughout the paper.

You state "These diseases also disproportionately affect socioeconomically disadvantaged populations, raising important equity considerations when designing carbon reduction strategies". I agree with the first half of the statement, but please clarify what is meant by the second half.

I understand why CVD and cancer were chosen for the study, but the rationale for RA and epilepsy over other conditions is unclear. For example chronic respiratory disease is more common with a higher morbidity and mortality.

Methodology

I note that you used the UK Government's 2021 Greenhouse Gas (GHG) Conversion Factors. These only include the use-phase emissions and take no account of vehicle production emissions. Given the relatively low proportion of patients who would already have access to electric cars, this may be an important issue as these vehicles do not currently exist. Sustainability relies on a net reduction in global carbon emissions and therefore I feel that philosophically we must consider where carbon emissions are simply moved rather than reduced.

How did you take into account the change of addresses over the study period?

I note that you simply looked at keeping the same number of journey miles but change the mode of transport. What about options to reduce the journey miles including moving specialist clinics into the community, primary care delivered services, remote consultations, improved education to reduce unnecessary visits, easy remote/telephone access to a clinician for ad hoc advice/support?

Just to be clear did you include data from all patients in the population with an index condition diagnosis within the study time frame? For general overarching conditions such as CVD and Cancer please clarify the actual diagnoses in scope. How did you define "without multimorbidity" as your control group? You can have any of your index conditions and still be "without multimorbidity"

Patient and public involvement

I think it is a shame not to have the PPI voice in this. Your proposed interventions are primarily the responsibility of patients and the public to implement and having information on feasibility, affordability and acceptability as well as an overview of whether these specific patient groups place value in carbon reduction, certainly enough to make any changes, is important. I note high levels of social deprivation and this can for example impact on access to more expensive technologies such as electric vehicles, however solutions such as remote consultations or closer to home may be more interesting for them.

Results

I note bus travel had “intermediate emissions”. Did you look at electric buses?

How were trains powered? How did you allow for travel to and from stations?

Electric public transport may be a good shout here as it solves the equity/personal affordability issue and adds value to society outside of the scope of health and care services.

Did you look at hospital provided transport?

Discussion

To what degree do you think these findings are generalisable to the UK population?

You have introduced discussion of other carbon reducing strategies but why not model them in your study? I am sure there are practical issues, for example the availability of data etc – but add this to the discussion.

Further telemed pilot in CVD intervention https://pubmed.ncbi.nlm.nih.gov/38562585/

Page 20: are treatment visits for radio or chemotherapy coded within the 87% of follow up visits? Are they not treatment visits?

Page 21: I am note sure I agree that “This underscores that one of the most effective strategies for reducing carbon emissions from patient travel (access to electric cars) extends beyond the direct control of the NHS”. Surely reducing the requirement to travel at all to bare minimum via remote medicine, education, patient support access and community based clinics/diagnostics are most effective and all within the direct control of NHS. An electric car on the drive with no need to go anywhere is not an effective carbon reduction strategy. Electrified NHS delivered patient transport could mop up any remaining necessary planned travel and again is in reach for the NHS to implement if this is deemed cost effective and sustainable. This also delivers equity of both access and sustainability.

VERSION 1 - AUTHOR RESPONSE

Reviewer 1

The study of Olsen et al. extimates the patient travel carbon emission across common chronic diseases and suggests carbon saving by alternative transport possibilities. To do end, data from 6,599 patients aged between 50 and 55 were ananlyzed retrospectively for three different periods. Cancer patients traveled the longest followed by RA patients. Travelling with electric vehicles and train have the lowest emission. To achieve a net-zero healthcare, the authors propose mulitsectoral strategies such as integrated transport planning and targeted telemedicine.

Even if the results are not suprising, they are stoll are important and should be of interest for a broad readership of BMJ Open. Some revisions should be made before publication.

Response:

We thank the reviewer for their positive comments on our manuscript and helpful suggestions for improvement. We have responded to each point in detail below.

General comment

1) Please explain all abbreviations when they are first used, including NHS.

Response:

The manuscript has been checked to ensure all abbreviations have been stated, including NHS.

2) The abstract provides a good overview about the study and the methods chapter is understandable.

Response:

Thank you for this feedback.

Results:

3) Table 2 contains a relatively large amount of data and is therefore confusing. Is all the data relevant for the readers in the main manuscript? Would it be possible to reduce the data in the main tables and include the detailed data in the Supplement?

Response:

We have reduced the information presented in Table 2, and now only present mean home-to-clinic distances by condition for the 5 time-periods. The full table, containing standard errors, medians and IQR, has been moved to the Supplementary Materials.

4) Starting on page 11 line 58 / 59: „Significant non-linear trends over time were observed for cancer, RA, and CVD (p<0.001), but not epilepsy (Figure 2).“ => What is the trend? It is rising or falling? The most important findings should be unterstable without looking at the figures and tables.

Response:

We have revised the text to better explain the trend. Updated text:

"Significant non-linear trends over time in travel distances were observed for cancer, RA, and CVD (p<0.001), which increased in the years up to diagnosis and then decreased in the years following, but not epilepsy (Figure 2)."

5) Figure 2: Consider a different structure for the figure, in which all graphs are displayed in a single line for each gender and the x-axes are not divided. One option is to arrange the data by high (women at the top, men at the bottom). All abbreviations should be explained in the figure legend (e.g. GEE...).

Response:

We have amended Figure 2 so that both males and females are plotted on the same graph for each condition. We agree this supports the interpretation and improves the flow of the manuscript. It also now clearly highlights no difference in values between males and females.

We have also explained the abbreviations in full in the figure legend.

6) The dicussion mainly advocates a greater focus on telemedicine. To support this hypothesis, you should calculate the carbon emission of one telemedine consultation compared to the various transport options.

Response:

We appreciate the reviewer's observation regarding our discussion of telemedicine. However, calculating carbon emissions for telemedicine consultations compared to various transport options falls outside the scope of the current study. Such an analysis

would require substantial methodological development and numerous assumptions that would warrant a dedicated investigation.

We acknowledge this as a valuable direction for future research and have incorporated the following recommendation into our discussion:

"We recommend future studies examine a range of clinical service delivery options associated with reducing patient carbon emissions, including moving specialist clinics into the community, primary care delivered services, remote consultations, improved education to reduce unnecessary visits, whilst considering its impact on outcomes, patient safety, technological availability and patient acceptability. Additionally, we suggest modelling the impact of these strategies on NHS carbon emissions."

Reviewer 2

I wish to congratulate the authors. This is an important topic and I find their work and the paper very interesting – I enjoyed reading it. I think this does add valuable insights and data into this debate. I think it would benefit from some revision. It reads a bit like an exercise to qualify travel and see how much CO2 we could save by making everyone have an electric car. The situation is more complex than this and the solutions much more wide ranging. The authors do expand to accept this, but quite late in the paper and this consideration is not apparent in the design or main substance. I really look forward to seeing where they could take this next and there is clear potential impact here.

Response:

We thank the reviewer for their positive comments on our manuscript and helpful suggestions for improvement. We have responded to each point in detail below.

Title

1) These data are very specific to the geography, health service design and healthcare use in a specific population: Consider reflecting this in the title and manuscript.

Response:

In response to this point, and that of the editor, we have revised the title of the manuscript to reflect the study design, setting and population.

The updated title "Transition towards healthcare 'net zero': Modelling condition-specific patient travel carbon emission estimations by transport mode: a retrospective population-based cohort study, Greater Glasgow, UK."

2) I think the title does underplay what you are trying to achieve here. You are looking at specific actionable opportunities to reduce emissions.

Response:

We have revised the title in response to this feedback. However, we have reservations about characterising our findings as actionable opportunities at this stage. As the reviewer notes in a subsequent comment, our study did not include patient and public involvement, and our findings represent potential opportunities that require further development before implementation. We now recommend in the discussion that additional consultation with patients, the public, and healthcare professionals is essential to translate these findings into truly actionable strategies. The evidence from this study provides a foundation to inform and support these necessary consultations.

Abstract

3) Participants: suggest listing cardiovascular first as largest group then in order of descending size followed by controls.

Response:

We have revised the order of conditions from largest (cardiovascular disease) to smallest, followed by controls.

Introduction

4) Page 6- You state "Cancer and cardiovascular disease (CVD) are leading causes of morbidity and mortality globally". Cardiovascular disease is both more common and a greater cause of morbidity and mortality than cancer – consider reversing the order here and throughout the paper.

Response:

We appreciate the reviewer's point regarding the epidemiological importance of cardiovascular disease relative to cancer. However, we have consistently listed these conditions in alphabetical order throughout the manuscript, which is a standard approach for reporting multiple clinical conditions of similar relevance to the study objectives.

Where we report outcomes that vary by magnitude, we have ordered them from largest to smallest to reflect their relative importance, and change the order in the abstract based on your earlier comment.

5) You state “These diseases also disproportionately affect socioeconomically disadvantaged populations, raising important equity considerations when designing carbon reduction strategies”. I agree with the first half of the statement, but please clarify what is meant by the second half.

Response:

We have expanded the sentence to provide meaning to the second half.

Updated sentence:

“These diseases also disproportionately affect socioeconomically disadvantaged populations, raising important equity considerations when designing carbon-reduction strategies as the burden of interventions or service delivery changes would have a significant impact on this population group”.

6) I understand why CVD and cancer were chosen for the study, but the rationale for RA and epilepsy over other conditions is unclear. For example chronic respiratory disease is more common with a higher morbidity and mortality.

Response:

The selection of rheumatoid arthritis and epilepsy was based on clinical expertise and their distinct care patterns, which provide valuable contrasts to cancer and cardiovascular disease. We chose rheumatoid arthritis because it requires regular, ongoing outpatient follow-up for disease monitoring, and epilepsy because it involves long-term specialist consultations that are typically more stable over time.

As stated in our introduction, we aimed to examine conditions with varying care intensity and frequency patterns:

“Rheumatoid arthritis is a chronic inflammatory condition necessitating regular outpatient follow-up for disease monitoring and management 16, offering insights into the cumulative travel burden associated with sustained care. Epilepsy, a long-term neurological disorder, also involves ongoing specialist consultations 17, albeit typically with less intensive resource use than cancer or CVD, providing a valuable contrast in travel-related emissions across different disease profiles.”

We acknowledge that other conditions, such as chronic respiratory disease, represent important areas for investigation given their prevalence and disease burden. We have added the following text to our discussion to address this limitation:

“The four clinical conditions included in the study were chosen based on consultation with clinicians to examine our outcomes across a range of contrasting care patterns. Future studies should explore these outcomes for a broader range of conditions, including those with higher population-level morbidity and mortality.”

Methodology

7) I note that you used the UK Government’s 2021 Greenhouse Gas (GHG) Conversion Factors. These only include the use-phase emissions and take no account of vehicle production emissions. Given the relatively low proportion of patients who would already have access to electric cars, this may be an important issue as these vehicles do not currently exist. Sustainability relies on a net reduction in global carbon emissions and therefore I feel that philosophically we must consider where carbon emissions are simply moved rather than reduced.

Response:

We appreciate the reviewer's important observation regarding the scope of our carbon emission calculations. The reviewer correctly identifies a significant limitation in using the UK Government's 2021 GHG Conversion Factors, which focus on use-phase emissions and do not account for the full lifecycle carbon footprint of vehicles, including manufacturing emissions.

This is particularly relevant given the current low adoption rates of electric vehicles among patients. As the reviewer notes, true sustainability requires consideration of whether carbon emissions are genuinely reduced versus simply relocated within the production and supply chain.

We have acknowledged this limitation by adding the following text to our limitations section:

"We utilised the UK Government's 2021 Greenhouse Gas (GHG) Conversion Factors to calculate emissions associated with patient journeys from home to clinic. However, these factors only capture use-phase emissions and do not include vehicle production emissions or other lifecycle factors associated with the transition to net zero. This may underestimate the true carbon impact of proposed interventions, particularly those involving electric vehicle adoption, where manufacturing emissions represent a substantial component of the total carbon footprint."

8) How did you take into account the change of addresses over the study period?

Response

We thank the reviewers for this question. In the revised methods section, we now clarify how we accounted for participants' changes of address during the study period. We have added the following text:

"For each patient's visit, the distance was calculated using the specific address listed in the patient's electronic health record at the time of that event. This ensures that any patient relocation during the study period is accurately reflected in our distance calculations"

9) I note that you simply looked at keeping the same number of journey miles but change the mode of transport. What about options to reduce the journey miles including moving specialist clinics into the community, primary care delivered services, remote consultations, improved education to reduce unnecessary visits, easy remote/telephone access to a clinician for ad hoc advice/support?

Response:

The reviewer raises an excellent point that represents a crucial area for future investigation. Our study focused on transport mode changes while maintaining existing service delivery patterns. We did not examine service delivery modifications such as community-based clinics, primary care integration, or remote consultations, as these interventions require comprehensive evaluation of their impact on clinical outcomes, patient safety, service quality, technological infrastructure, and patient acceptability.

We acknowledge this important limitation and have addressed it in our discussion with the following enhanced paragraph:

"The NHS Scotland Climate Emergency and Sustainability Strategy outlines plans to minimise patient and visitor travel through adoption of 20-minute neighbourhood

principles in health facility planning and promotion of integrated care models to reduce separate appointments and associated journeys. However, this approach may conflict with evidence supporting high-quality specialist centres, which have demonstrated improved patient outcomes. Furthermore, only 51% of Scottish households are within a 10-minute walking distance of a community healthcare centre, highlighting accessibility challenges.

Additional barriers include technological infrastructure requirements, particularly reliable internet connectivity and accessible devices in rural or underserved regions. Digital literacy barriers, reimbursement concerns, and user acceptance must also be addressed to facilitate telemedicine adoption. Long-term research is needed to confirm telemedicine's efficacy across diverse clinical scenarios and identify potential limitations.

We recommend future studies examine a range of clinical service delivery options that could reduce patient carbon emissions, including relocating specialist clinics to community settings, expanding primary care-delivered services, implementing remote consultations, and improving patient education to reduce unnecessary visits. Such investigations should consider the impact on clinical outcomes, patient safety, technological accessibility, and patient acceptability. Additionally, we suggest modelling the carbon emission implications of these strategies for the NHS as a whole."

10) Just to be clear did you include data from all patients in the population with an index condition diagnosis within the study time frame? For general overarching conditions such as CVD and Cancer please clarify the actual diagnoses in scope. How did you define "without multimorbidity" as your control group? You can have any of your index conditions and still be "without multimorbidity"

Response:

We appreciate the reviewer's insightful question regarding the precise scope of the overarching conditions as well as the control group. We have updated the methods section to more clearly define each study condition and the control group. The following text has been added:

"Patients were included if they were diagnosed with one of four selected conditions, cancer, cardiovascular disease (CVD), rheumatoid arthritis (RA), or epilepsy, during the diagnosis window, and had no other comorbidity. This specific diagnosis window ensured each patient had the required two-year pre- and post-diagnosis data for analysis. The precise diagnostic criteria for RA and epilepsy, along with the specific diseases included under the umbrella terms of cancer and CVD, were ascertained

through either an eligible ICD-10 diagnosis code recorded in SMR01 or eligible BNF-coded medication recorded in the PIS (Supplementary Table 1). For each condition, the date of first diagnosis was defined as the earliest date on which an eligible diagnosis or medication code was recorded. All patients within the eligible study population who received an index diagnosis for the studied conditions during the defined study time frame were included in this study. For each patient with an index condition, a corresponding control participant without the condition was selected from the same population. This control cohort was established using an exact matching methodology, pairing individuals based on age, sex, and their home SIMD quintile. To ensure comparable observation periods, the diagnosis year of the case patient was assigned as the index year for their reference comparison patient."

Patient and public involvement

11) I think it is a shame not to have the PPI voice in this. Your proposed interventions are primarily the responsibility of patients and the public to implement and having information on feasibility, affordability and acceptability as well as an overview of whether these specific patient groups place value in carbon reduction, certainly enough to make any changes, is important. I note high levels of social deprivation and this can for example impact on access to more expensive technologies such as electric vehicles, however solutions such as remote consultations or closer to home may be more interesting for them.

Response:

We agree with the reviewer, and in response to earlier points we have highlighted that patient consultation is required for carbon reducing solutions.

Results

12) I note bus travel had "intermediate emissions". Did you look at electric buses?

Response:

We did not specifically examine electric buses in our analysis. Our calculations were based on the UK Government's 2021 Greenhouse Gas (GHG) Conversion Factors, which provide emissions data for buses categorised as either "London" or "non-London local bus" services. Given that our study was conducted in Glasgow, we applied the "non-London local bus" conversion factor.

The reviewer's question highlights an important limitation, as electric buses are increasingly being deployed across UK cities, including Glasgow, and would have significantly lower operational emissions than conventional diesel buses. This represents another example of how our use of standard conversion factors may not capture the evolving transport landscape.

We have incorporated this limitation into our discussion:

"Limitations include the absence of individual-level data on actual transport modes, fuel type, or specific patient behaviours (e.g. trip chaining or caregiver travel), which limits precision in estimating emissions. Although we modelled a range of modal scenarios, these remain assumptions in the absence of travel survey validation. We utilised the UK Government's 2021 Greenhouse Gas (GHG) Conversion Factors to calculate emissions associated with patient journeys from home to clinic. However, these factors only capture use-phase emissions and do not include vehicle production emissions or other lifecycle factors associated with the transition to net zero. Additionally, the standard conversion factors may not reflect local variations in fleet composition, such as the increasing deployment of electric buses in urban areas. This may underestimate the true carbon impact of proposed interventions, particularly those involving electric vehicle adoption, where manufacturing emissions represent a substantial component of the total carbon footprint."

13) How were trains powered? How did you allow for travel to and from stations?

Response:

As above, we used the UK Government's 2021 Greenhouse Gas (GHG) Conversion Factors for local trains which do not estimate whether this was electric or diesel.

As our study already makes a number of assumptions in terms of the travel mode and home to clinic distances, we did not allow in our analysis for travel to and from public transit stops. This is a sophisticated analysis that has a number of assumptions in itself in terms of which transit stop would be chosen, frequency of transit visits to stop, and number multi trips.

We have added the limitation to the discussion:

"Our analysis also does not account for the journey to or from a public transit stop that may be on foot and assumes the whole journey was by a transport single mode."

14) Electric public transport may be a good shout here as it solves the equity/personal affordability issue and adds value to society outside of the scope of health and care services.

Response:

We strongly agree with the reviewer's observation about the broader societal benefits of electric public transport. This approach addresses both carbon reduction and health equity concerns, as it removes individual financial barriers to accessing lower-emission transport while providing community-wide environmental and mobility benefits that extend well beyond healthcare services.

This important point is reflected in our discussion, where we acknowledge both the potential and the implementation challenges:

"This highlights that effective strategies for reducing carbon emissions from patient travel requires multi-sectoral approaches—including the NHS and government-led initiatives."

We also emphasize the need for broader system-level approaches:

"Achieving a net-zero healthcare system demands more than technical solutions—it calls for system-wide innovation, political will, and the embedding of environmental sustainability into clinical care pathways. By quantifying condition-specific travel emissions, this study offers a foundation for targeted action, supporting a greener NHS that maintains high-quality, accessible care for all."

The reviewer's point reinforces our conclusion that meaningful carbon reduction in healthcare-related transport requires coordinated action across sectors, with electric public transport representing a particularly promising intervention that simultaneously addresses environmental, equity, and accessibility objectives.

15) Did you look at hospital provided transport?

Response:

We did not examine hospital-provided transport in this study. During initial discussions with NHS Greater Glasgow and Clyde, we found that the health board does not systematically record patient travel data, conduct patient travel surveys, or collect personal information linking hospital-provided transport users to specific clinical conditions.

This represents a data limitation that prevented us from including this transport mode in our analysis. Hospital-provided transport could represent an important component of patient travel emissions and may serve patients with specific mobility needs or those from areas with limited public transport access.

We acknowledge this as a limitation that future studies should address, potentially through dedicated data collection protocols that could capture the emissions profile and patient demographics associated with hospital-provided transport services.

Discussion

16) To what degree do you think these findings are generalisable to the UK population?

Response:

Our findings are likely generalizable to other major UK urban areas, given that our study encompasses NHS Greater Glasgow and Clyde, which serves a substantial urban population of 1.3 million people. However, generalisability may be more limited for rural or smaller urban settings, where transport infrastructure, service accessibility, and patient travel patterns may differ significantly.

As stated in our discussion:

"This study draws on a comprehensive, population-level cohort from NHS Greater Glasgow and Clyde, representing a population of 1.3 million people, meaning the results are likely generalisable to other major urban areas in the UK."

17) You have introduced discussion of other carbon reducing strategies but why not model them in your study? I am sure there are practical issues, for example the availability of data etc – but add this to the discussion.

Response:

The reviewer raises an important methodological question about why we discussed but did not model alternative carbon reduction strategies such as service reconfiguration, telemedicine, or community-based care delivery.

While these interventions represent promising avenues for emission reduction, modelling them was beyond the scope of our current study due to several practical

constraints. These strategies would require comprehensive data on service delivery patterns, patient outcomes, technological infrastructure, and acceptability that are not readily available in existing datasets. Additionally, such modelling would necessitate complex assumptions about implementation scenarios, uptake rates, and potential unintended consequences that would warrant dedicated research protocols.

We acknowledge this as an important limitation and have addressed it in our discussion by recommending future research directions:

"We recommend future studies examine a range of clinical service delivery options that could reduce patient carbon emissions, including relocating specialist clinics to community settings, expanding primary care-delivered services, implementing remote consultations, and improving patient education to reduce unnecessary visits. Such investigations should consider the impact on clinical outcomes, patient safety, technological accessibility, and patient acceptability. Additionally, we suggest modelling the carbon emission implications of these strategies for the NHS as a whole."

18) Further telemed pilot in CVD intervention https://pubmed.ncbi.nlm.nih.gov/38562585/

Response:

Thank you, we have added this reference to the discussion.

19) Page 20: are treatment visits for radio or chemotherapy coded within the 87% of follow up visits? Are they not treatment visits?

Response:

We thank the reviewers for their insightful question. Our analysis included all scheduled clinic visits and inpatient admissions that occur after the initial index diagnosis. We have expanded method section to clarify that the follow-up visits included visits where patients received active treatment, such as radiotherapy or chemotherapy. The following text has been inserted:

"The included visits consisted of all scheduled clinic appointments and inpatient admissions after the initial diagnosis, covering periods of active treatment, such as radiotherapy and chemotherapy, as well as attendances for routine monitoring".

20) Page 21: I am note sure I agree that “This underscores that one of the most effective strategies for reducing carbon emissions from patient travel (access to electric cars) extends beyond the direct control of the NHS”. Surely reducing the requirement to travel at all to bare minimum via remote medicine, education, patient support access and community based clinics/diagnostics are most effective and all within the direct control of NHS. An electric car on the drive with no need to go anywhere is not an effective carbon reduction strategy. Electrified NHS delivered patient transport could mop up any remaining necessary planned travel and again is in reach for the NHS to implement if this is deemed cost effective and sustainable. This also delivers equity of both access and sustainability.

Response:

We appreciate the reviewer's thoughtful critique of our statement and agree with their fundamental point. The reviewer correctly identifies that the most effective carbon reduction strategies—eliminating unnecessary travel through service reconfiguration, remote consultations, patient education, and community-based care—are indeed within NHS control and should be prioritised over solutions that maintain existing travel patterns, but also requite multi-sectoral support.

We have revised our discussion to better reflect this perspective:

“This highlights that effective strategies for reducing carbon emissions from patient travel requires multi-sectoral approaches—including the NHS and government-led initiatives.”

VERSION 2 - REVIEW

**Reviewer**	**1**
**Name**	**Kühl, Susanne**
**Affiliation**	**Ulm University, Biochemistry and Molecular Biology**
**Date**	**08-Oct-2025**
**COI**	

Table 2:

Time points should be defined in the table legend

In wich column the km were shown? This should clearly be explained in the legend.

Table 3:

The legend is extremely short. The content of the table plus legend should be self-explaining without reading the main text.

**Reviewer**	**2**
**Name**	**Carradice, Daniel**
**Affiliation**	**Hull York Medical School**
**Date**	**02-Oct-2025**
**COI**	

For disclosure – I was previously "reviewer 2".

I would congratulate the authors in responding favourably to feedback and have in my view improved the quality of the manuscript. Well done. I stand by m original comments and feel that this paper adds to the existing literature and builds a conversation towards how we can or perhaps should tackle these issues.

I have a few niggling comments I am afraid.

I remain unsure about your use of the term "without multimorbidity" to describe your "healthy controls". I would prefer the latter term and define it. Many of the patients in your disease groups will be without multimorbidity too.

In the abstract I don't think you need to labour the fact that the cancer patients have the highest emissions – you have already stated that they travel the furthest distance and unless you know precisely what everyone's mode of transport was you are simply basing emissions on distance.

In the abstract you haven't included your bus results.

What do you mean by "Condition-specific patterns in healthcare-related travel emissions provide actionable insight into decarbonising healthcare"? I am not sure that the condition specific aspect of this work translates into actionable insight. Are differences in travel distances between them relevant unless you are able to look at pathway specific optimisation strategies? Are you simply getting a range of estimates for the size of the problem/opportunity in a mixed healthcare-seeking population?

VERSION 2 - AUTHOR RESPONSE

Reviewer 1

Table 2:

Time points should be defined in the table legend

In wich column the km were shown? This should clearly be explained in the legend.

Response:

We have updated the column titles to state what the values show, this has been update from ‘female’ to ‘Mean total annual distances travelled (km): female’.

We have now defined the time points in the table legend. The following text has been added: “Time points: -2 = 2-years pre-diagnosis, –1 = 1-year pre-diagnosis, 0 = diagnosis year, 1 = 1-year post-diagnosis, 2 = 2-years post-diagnosis.”

Table 3:

The legend is extremely short. The content of the table plus legend should be self-explaining without reading the main text.

Response:

We have updated the Table header and added additional notes:

Updated legend: “Associations between annual distances travelled by time-point (pre and post diagnosis), and condition, controlling for distance from home and deprivation”

Updated note: “The table examines annual distances travelled by condition, time-point and patients’ residential area-level deprivation, as well as interactions between these. Time point (in years) was treated as a continuous variable and included as a second order polynomial to allow for a non-linear association with distance.”

Reviewer 2

I would congratulate the authors in responding favourably to feedback and have in my view improved the quality of the manuscript. Well done. I stand by m original comments and feel that this paper adds to the existing literature and builds a conversation towards how we can or perhaps should tackle these issues.

I have a few niggling comments I am afraid.

I remain unsure about your use of the term “without multimorbidity” to describe your “healthy controls”. I would prefer the latter term and define it. Many of the patients in your disease groups will be without multimorbidity too.

Response:

We agree with this comment and have renamed the 'without multimorbidity' group '**control group'**. We have not called the group 'healthy' controls as they were matched based on being without multimorbidity and the conditions named, but their health status is unknown.

We have provided the description of the group in the methods.

"A matched control group was also created: for each patient with an index condition, a corresponding control participant without the condition was selected from the same population. This control group was established using an exact matching methodology, pairing individuals based on age, sex, and their home SIMD quintile. To ensure comparable observation periods, the diagnosis year of the case patient was assigned as the index year for their reference comparison patient."

The abstract has been updated to define this:

"6,599 patients aged 50 to 55 years at diagnosis, including cardiovascular disease (CVD; n=1,711), epilepsy (n=1,044), cancer (n=716), rheumatoid arthritis (RA; n=172), and a matched control group based on age, sex, and area-level deprivation (n=2,956)"

The labelling throughout the text, figures, tables and supplementary materials have been amended.

In the abstract I don't think you need to labour the fact that the cancer patients have the highest emissions – you have already stated that they travel the furthest distance and unless you know precisely what everyone's mode of transport was you are simply basing emissions on distance.

Response:

We have revised the abstract to reduce the focus on cancer patients.

Change 1:

original text "Assuming 100% petrol car use, cancer patients produced the highest emissions: 16.5 kg CO_2/patient/year , followed by RA (12.5 kg), CVD (10.5 kg), and epilepsy (to 8.0 kg).",

revised text "Assuming 100% petrol car use, estimated condition specific emissions ranged from: 16.5 kg CO_2/patient/year to 8.0 kg.".

Change 2:

original text "When travel was modelled using electric vehicles, emissions dropped to 3.5 kg for cancer, and below 2.7 kg for all other conditions"

revised text "When travel was modelled using electric vehicles, emissions dropped between 3.5 kg to 2.7 kg for all conditions."

Change 3:

original text "Reducing petrol car travel from 100% to 60% among cancer patients lowered emissions by 6.6 kg CO_2/patient"

revised text "Reducing petrol car travel from 100% to 60% lowered emissions up to 6.6 kg CO_2/patient."

In the abstract you haven't included your bus results.

Response:

We have added the following result for bus travel to the abstract:

"Bus travel resulted in intermediate emissions, estimated between10.5 to 8.0 kg CO_2/patient".

What do you mean by "Condition-specific patterns in healthcare-related travel emissions provide actionable insight into decarbonising healthcare"? I am not sure that the condition specific aspect of this work translates into actionable insight. Are differences in travel distances between them relevant unless you are able to look at pathway specific optimisation strategies? Are you simply getting a range of estimates for the size of the problem/opportunity in a mixed healthcare-seeking population?

Response:

We have revised this sentence to highlight that the condition-specific estimates provide baseline understanding of the opportunities and challenges for decarbonising healthcare.

Updated abstract text:

"Condition-specific estimates of healthcare-related travel emissions provide baseline understanding of the opportunities and challenges for decarbonising healthcare."
